# Searching for Genetic Biomarkers for Hereditary Angioedema Due to C1-Inhibitor Deficiency (C1-INH-HAE)

**DOI:** 10.3389/falgy.2022.868185

**Published:** 2022-07-07

**Authors:** Faidra Parsopoulou, Gedeon Loules, Maria Zamanakou, Dorottya Csuka, Agnes Szilagyi, Maria Kompoti, Grzegorz Porebski, Fotis Psarros, Markus Magerl, Anna Valerieva, Maria Staevska, Krystyna Obtulowicz, Marcus Maurer, Matthaios Speletas, Henriette Farkas, Anastasios E. Germenis

**Affiliations:** ^1^Department of Immunology and Histocompatibility, Faculty of Medicine, School of Health Sciences, University of Thessaly, Larissa, Greece; ^2^CeMIA SA, Larissa, Greece; ^3^Department of Internal Medicine and Haematology, Hungarian Angioedema Center of Reference and Excellence, Semmelweis University, Budapest, Hungary; ^4^Department of Clinical and Environmental Allergology, Jagiellonian University Medical College, Krakow, Poland; ^5^Department of Allergology, Navy Hospital, Athens, Greece; ^6^Institute of Allergology, Charité – Universitätsmedizin Berlin, Freie Universität Berlin and Humboldt-Universität zu Berlin, Berlin, Germany; ^7^Fraunhofer Institute for Translational Medicine and Pharmacology ITMP, Allergology and Immunology, Berlin, Germany; ^8^Department of Allergology, Clinic of Allergology, University Hospital “Alexandrovska”, Medical University of Sofia, Sofia, Bulgaria

**Keywords:** C1-inhibitor deficiency, genetic biomarkers, functional variants, hereditary angioedema, long-term prophylaxis, next-generation sequencing, severity score

## Abstract

Existing evidence indicates that modifier genes could change the phenotypic outcome of the causal *SERPING1* variant and thus explain the expression variability of hereditary angioedema due to C1-inhibitor deficiency (C1-INH-HAE). To further examine this hypothesis, we investigated the presence or absence of 18 functional variants of genes encoding proteins involved in the metabolism and function of bradykinin, the main mediator of C1-INH-HAE attacks, in relation to three distinct phenotypic traits of patients with C1-INH-HAE, i.e., the age at disease onset, the need for long-term prophylaxis (LTP), and the severity of the disease. Genetic analyses were performed by a validated next-generation sequencing platform. In total, 233 patients with C1-INH-HAE from 144 unrelated families from five European countries were enrolled in the study. Already described correlations between five common functional variants [*F12*-rs1801020, *KLKB1*-rs3733402, *CPN1*-rs61751507, and two in *SERPING1* (rs4926 and rs28362944)] and C1-INH-HAE severity were confirmed. Furthermore, significant correlations were found between either the age at disease onset, the LTP, or the severity score of the disease and a series of other functional variants (*F13B*-rs6003, *PLAU*-rs2227564, *SERPINA1*-rs28929474, *SERPINA1*-rs17580, *KLK1*-rs5515, *SERPINE1*-rs6092, and *F2*-rs1799963). Interestingly, correlations uncovered in the entire cohort of patients were different from those discovered in the cohort of patients carrying missense causal *SERPING1* variants. Our findings indicate that variants other than the *SERPING1* causal variants act as independent modifiers of C1-INH-HAE severity and could be tested as possible prognostic biomarkers.

## Introduction

Hereditary angioedema due to C1-inhibitor deficiency (C1-INH-HAE), an autosomal dominant disorder with recurrent attacks of edema spontaneously developing in any body location, is characterized by a large heterogeneity in its clinical expression, including the age at disease onset, the number and triggers of attacks, the severity and localization of edema, and prodromal signs and symptoms (1, 2). These features show variable expressivity, i.e., it may vary even among members of the same family carrying the same *SERPING1* causal mutation, which, at present, is unpredictable and largely unexplained.

Accumulating evidence indicates that modifier genes could change the phenotypic outcome of the variant at the primary mutation in the target *SERPING1* gene, and thus explain disease expression variability (3). Understanding genetic modification phenomena will obviously improve our ability to better manage the disabling and potentially fatal manifestations of C1-INH-HAE. To this aim, we investigated here the relationship between parameters associated with disease course severity and common functional variants in genes involved in the metabolism of bradykinin, the main mediator of angioedema attacks in patients with C1-INH-HAE.

## Patients and Methods

This study included 233 patients (104 men and 129 women, mean age 40 years, range 2.5–85) with C1-INH-HAE (217 type I, 16 type II) from 144 unrelated families (16 Bulgarian, 23 German, 30 Greek, 43 Hungarian, and 32 Polish). All patients were previously genotyped for *SERPING1* mutations. Demographic, clinical, and molecular data of the patients are presented in Table 1. Patients' medical records were reviewed and data regarding the age at disease onset and the long-term prophylaxis (LTP) were recorded. For patients not receiving long-term prophylactic treatment, the severity score Cutaneous Abdominal Laryngeal Score (CALS) was calculated according to the equation “CALS=1^*^Cutaneous+2^*^Abdominal+3^*^Laryngeal last year attacks”.

**Table 1:** Demographic, clinical, and molecular data of the C1-INH-HAE patients.

**Clinical data**	**Total**	**Greek**	**Polish**	**German**	**Hungarian**	**Bulgarian**
No (patients, families)	233, 144	31, 30	47, 32	23, 23	113, 43	19, 16
Sex (male/female)	104/129	17/14	18/29	11/12	48/65	10/9
Age at analysis (median, range)	40.0 (2.5–85)	35.0 (2.5–67)	44.0 (25–85)	42.0 (13–81)	39.0 (9–82)	47.0 (8–81)
Age at onset (median, range)	12.5 (0.5–73)	9.0 (1–31)	17.0 (1–73)	10.0 (3–19)	12.0 (0.5–53)	11.0 (1–50)
HAE Type (I/II)	217/16	31/0	44/3	22/1	102/11	18/1
Longterm treatment (Yes/No/NA)	79/141/13	11/16/4	3/42/2	6/10/7	55/58/0	4/15/0
CALS Severity (median, range)	26.74 (0–238)	24.72 (4–69)	43.95 (0–238)	Missing data	10.74 (0–88)	41.11 (6–103)
***SERPING1*** **defects**						
Regulatory, n (%)	1 (0.43%)	0 (0.00%)	0 (0.00%)	1 (4.35%)	0 (0.00%)	0 (0.00%)
Missense mutations, n (%)	69 (29.61%)	6 (19.36%)	20 (42.55%)	8 (34.78%)	33 (29.20%)	2 (10.53%)
Nonsense mutations, n (%)	33 (14.16%)	5 (16.13%)	5 (10.64%)	2 (8.70%)	17 (15.04%)	4 (21.05%)
Splice defects, n (%)	22 (9.44%)	4 (12.90%)	1 (2.13%)	3 (13.04%)	12 (10.62%)	2 (10.53%)
Small deletions or insertions (frameshift alterations), n (%)	46 (19.74%)	11 (35.48%)	2 (4.26%)	6 (26.08%)	24 (21.24%)	3 (15.78%)
Large deletions or insertions, n (%)	28 (12.02%)	1 (3.23%)	7 (14.89%)	2 (8.70%)	16 (14.16%)	2 (10.53%)
Deep intronic, n (%)	2 (0.86%)	2 (6.45%)	0 (0.00%)	0 (0.00%)	0 (0.00%)	0 (0.00%)
Unidentified defects, n (%)	32 (13.74%)	2 (6.45%)	12 (25.53%)	1 (4.35%)	11 (9.74%)	6 (31.58%)

DNA samples were analyzed in a validated next-generation sequencing (NGS) platform (Ampliseq custom panel, Thermo Scientific, Waltham MA, USA), as previously described (4, 5). Briefly, DNA libraries were constructed for each sample using the Ion AmpliSeq Library Kit 2.0 (Thermo Scientific) and indexed with a unique adapter using the Ion Xpress barcode adapter kit (Thermo Scientific). Template preparation, enrichment, and chip loading were carried out on the Ion Chef System (Thermo Scientific). Sequencing was performed on S5XL on 520 and 530 chips, using the Ion 510, Ion 520, and Ion 530 Kit-Chef (Thermo Scientific). All procedures were performed according to the manufacturer's instructions. Base calling, demultiplexing, and alignment to the hg19 reference genome (GRCh37) of the raw sequencing data were performed in Torrent Suite 5.10 software (Thermo Scientific, Waltham, MA, USA) using the default parameters. Variant calling was performed by the VariantCaller v.5.8.0.19 plug-in and coverage analysis by the CoverageAnalysis v.5.8.0.8 plug-in in Torrent Suite 5.10. All variants were annotated on Ion Reporter Software (Thermo Scientific).

We investigated the presence or absence of 18 common functional variants (allele frequency ≥1%) in relation to three distinct phenotypic traits of patients, i.e., the age at disease onset, the need for LTP, and the severity of the disease based on the CALS score. The investigated variants were variants of genes encoding proteins involved in the metabolism and function of bradykinin, the main mediator of C1-INH-HAE attacks, which were chosen based on their effect on protein activity, their frequency, and the coverage that could be achieved by the platform (Table 2). The local institutional review boards approved this study, and written informed consent was obtained from each individual or an accompanying relative.

**Table 2:** Selected common functional variants.

**Gene**	**Protein**	**dbSNP**	**Nucl. change**	**aa change**	*OMIM	**References**
*F12*	Factor XII	rs1801020	c.-4T>C	NA (5'UTR)	610,619	(6–9)
*F13A1*	Factor XIII Subunit A	rs5985	c.103G>T	p.Val35Leu	134,570	(10–13)
*F13B*	Factor XIII Subunit B	rs6003	c.344G>A	p.Arg115His	134,580	(14)
*F2*	Factor II	rs1799963	c.*97G>A	NA (3'UTR)	176,930	(15, 16)
*CPN1*	Carboxypeptidase N	rs61751507	c.533G>A	p.Gly178Asp	603,103	(17, 18)
*A2M*	Alpha-2-Macroglobulin	rs669	c.2998A>G	p.Ile1000Val	103,950	(19)
*KLK1*	Kallikrein 1	rs5515	c.230G>A	p.Arg77His	147,910	(20, 21)
*KLKB1*	Plasma Kallikrein B (Prekallikrein)	rs3733402	c.428G>A	p.Ser143Asn	229,000	(22)
*MASP2*	Mannan-binding lectin serine protease 2	rs72550870	c.359A>G	p.Asp120Gly	605,102	(23, 24)
*MPO*	Myeloperoxidase	rs56378716	c.752T>C	p.Met251Thr	606,989	(25, 26)
*PLAU*	Urinary plasminogen activator (urokinase, plasminogen activator)	rs2227564	c.422T>C	p.Leu141Pro	191,840	(27–29)
*SERPINA1*	Serine protease inhibitor, clade a, member 1 (a1-antitrypsin)	rs28929474	c.1096G>A	p.Glu366Lys	107,400	(30–36)
		rs17580	c.863A>T	p.Glu288Val		(37–40)
		rs121912714	c.839A>T	p.Asp280Val		(41–43)
*SERPINE1*	Serine protease inhibitor, clade e, member 1 (Nexin, PAI-1)	rs6092	c.43G>A	p.Ala15Thr	173360	(44)
*TLR2*	Toll-like receptor 2	rs5743708	c.2258G>A	p.Arg753Gln	603028	(45)
*SERPING1*	Serine protease inhibitor, clade g, (C1-INH)	rs28362944	c.-21T>C	NA (5'UTR)	606860	(46–52)
		rs4926	c.1438G>A	p.Val480Met		(53–55)

## Statistical Analysis

Categorical variables were analyzed with Fisher's exact test. Normality of continuous variables was assessed with Kolmogorov–Smirnov test. Normally distributed data were analyzed with Student's *t*-test and one-way ANOVA as appropriate. Skewed data were analyzed with nonparametric methods (Mann–Whitney test or Kruskal–Wallis test as appropriate). Given the fact that our patient population consisted of correlated subjects (members of individual families), we implemented generalized estimating equations (GEE), an extension of the generalized linear model that accounts for the within-subject correlation. GEE was used to model the relationship between explanatory variables (polymorphisms, sex, etc.) and response variables (age at disease onset, need for long-term treatment, CALS severity). Age at disease onset and CALS severity score were modeled as continuous variables in linear GEE models and the need for long-term treatment was entered as a binary variable in logistic GEE models. In all GEE models, an unstructured correlation structure was used, and the Quasi-Likelihood Information Criterion (QIC) was used for model selection. Data analysis was performed with SPSS 17.0 (IBM Corporation, NY, 2008). For all analyses, alpha was set at 0.05 (two-tailed). In cases in which multivariable analysis was more appropriate, such analysis was performed with the dependent variable, including the age at disease onset, the LTP or the CALS, and with those of the variants presenting significant associations in univariable analysis fitted as independent variables. This type of analysis was performed for two groups of C1-INH-HAE patients (a) independently of the *SERPING1* variant and (b) for patients carrying a missense variant in *SERPING1* (*n* = 69). The common functional variants *SERPINA1*-rs121912714, *SERPINA1*-rs28929474, and *MPO*-rs56378716 were not detected in any patient of the missense group.

## Results and Discussion

The allele frequency of the selected SNPs in our cohort did not differ significantly from the Global Allele Frequency (GMAF) and the European Non-Finnish Allele Frequency (ENFMAF) as recorded by GnomAD v2.1.1 (Supplementary Table 1). Similarly, the prevalence of the polymorphisms did not differ significantly between examined patient groups from different countries (Supplementary Table 2).

The correlations found between functional variants and the age at disease onset, the LTP, or the severity score of the disease are summarized in Table 3. Five common functional variants had been previously correlated with C1-INH-HAE severity – *F12*-rs1801020, *KLKB1*-rs3733402, *CPN1*-rs61751507, and two in *SERPING1* (rs4926 and rs28362944).

**Table 3:** Summary of the correlations of common functional variants with patients' phenotype – age at disease onset, need for LTP, CALS severity score.

	**SNP *(Gene)***	**Nucl. change**	**Genotype**	**Age at disease onset**		**Need for LTP**		**CALS**	
All *SERPING1* variants	*SERPING1*-rs4926	c.1438G>A	GA AA	+3.6 years +6.3 years	*p* = 0.018 *p* = 0.058	– –		– –	
	*SERPING1*-rs28362944	c.-21T>C	TC and CC	–		2.5-fold	*p* = 0.012	–	
	*F12*-rs1801020	c.-4T>C	CT CC	– –		– –		+18.69 +28.21	*p* = 0.002 *p* < 0.001
	*F13B*-rs6003	c.344G>A	GA	–		–		−11.84	*p* = 0.024
	*SERPINA1-*rs28929474	c.1096G>A	GA and AA	–		–		+80.16	*p* = 0.003
	*PLAU*-rs2227564	c.422T>C	TT	–		–		−13.67	*p* = 0.004
Missense *SERPING1* variants	*SERPINA1-*rs17580	c.863A>T	AT	−8 years	*p* < 0.001	–		–	
	*KLKB1-*rs3733402	c.428G>A	AA	−7 years	*p* = 0.029	–		+30.45	*p* < 0.001
	*KLK1-*rs5515	c.230G>A	GA	+8.95 years	*p* = 0.05	–		−16.79	*p* = 0.029
	*SERPINE1-*rs6092	c.43G>A	AA	+8.4 years	*p* = 0.009	–		–	
	*SERPING1-*rs28362944	c.-21T>C	TC	–		4.2-fold	*p* = 0.02	–	
	*CPN1-*rs61751507	c.533G>A	GA	–		98% probability decrease	*p* = 0.017	–	
	*F12-*rs1801020	c.-4T>C	CT CC	+5 years CT *comp. to CC*	*p* < 0.001	– –	– –	+13.88 +25.48	*p* = 0.003 *p* = 0.002
	*F2-*rs1799963	c.*97G>A	GA	–		–		−25.97	*p* = 0.017

The presence of the C allele of the *F12*-rs1801020 (c.-4T>C) was significantly correlated with an increase in disease severity. More precisely, independently of the type of *SERPING1* mutations, homozygotes (CC) and heterozygotes of this variant present a mean disease severity score higher by 28.21 (*p* < 0.001) and 18.69 (*p* = 0.002) units of the CALS severity score, respectively. Similarly, homozygotes (CC) and heterozygotes (CT) of this variant carrying *SERPING1* missense variants present a mean disease severity score higher by 25.48 (*p* = 0.002) and by 13.88 (*p* = 0.003) units of the CALS severity score, respectively, compared with CALS score in patients lacking the polymorphism. Our results agree with evidence provided by Bors et al. (6) who suggested that the carriage of the T allele of the *F12*-rs1801020 variant is independently associated with a less severe C1-INH-HAE clinical phenotype. Moreover, this result is in agreement with Rijavec et al. (7) who have shown that the C allele and the CC genotype were represented more in symptomatic patients, compared to asymptomatic. These authors suggested that carriers of the CC genotype have a 25-fold greater risk of developing the disease compared to those carrying the TT genotype. In our study, *F12*-rs1801020 displays a robust linear trend among the ordinal categories (homozygosity-heterozygosity-absence), indicating that the T allele provides a protective effect in regards to angioedema severity. Moreover, univariable analysis in patients with missense *SERPING1* mutations uncovered a significant 5-year delay at disease onset in heterozygous (TC) compared to homozygous (CC) patients (*p* < 0.001), a result that agrees with our previous findings (8). These effects could be explained by the findings of Kanaji et al. (9). These authors observed different levels of FXII in plasma, depending on the genotype. Even though both alleles were equally transcribed in hepatocytes of heterozygotes, the cDNA containing the T allele was producing less FXII *in vitro* than the one containing the C allele. Therefore, the presence of the variant is affecting the efficiency of translation.

In regards to the carriage of the C allele of the *SERPING1*-rs28362944 (c.-21T>C) variant, the probability of the need for LTP was found increased by 4.2-fold (*p* = 0.02) and 2.5-fold (*p* = 0.012) among patients with C1-INH-HAE who were carrying missense *SERPING1* mutations and independently of the *SERPING1* variation, respectively. The variant had been previously characterized as likely pathogenic when in a homozygous state (49, 50), despite that no correlation between this variant and the biochemical values of C1-INH function or the clinical severity score has been reported by other investigators (51). Interestingly, however, Duponchel et al. (52) have proposed the variant as a modifier of disease severity as they had found that the variant yields low but significant levels of exon 2 skipping in transfected cells. Therefore, this allele may contribute, at the RNA level, to more severe forms of C1-INH-HAE. In accordance, Cumming et al. (54) reported an increased disease penetrance in carriers of c.-21T>C when the variant allele presented in *trans* with the *SERPING1* mutation. Unfortunately, segregation analysis in our cohort could not be performed in many cases due to a lack of available family members.

The *SERPING1*-rs4926 (c.1438 G>A, p.Val480Met) had been predicted as deleterious and possibly damaging according to bioinformatic tools, because the highly conserved amino acid (Val) is important for the folding of the C1-INH protein into its native conformation (53). However, it is a well-documented common variant and characterized by different groups as benign in public databases. Independently of the *SERPING1* causal mutation, a significant 3.6-year (*p* = 0.018) and a trend toward 6.3-year (*p* = 0.058) delay at the age of disease onset was found in heterozygous (GA) and homozygous (AA) carriers, respectively. Functional studies by Cumming et al. (54) found no detectable effect of this variant on C1-INH structure, function, stability, plasma levels, or disease expression. However, the authors did not exclude a consequence of the variant on other functions of C1-INH as a modulator of the coagulation and kinin release pathways.

Homozygosity (AA) for *KLKB1*-rs3733402 (c.428G>A, p.Ser143Asn) in carriers of a missense *SERPING1* variant was significantly associated with 7-year earlier disease onset (*p* = 0.029) and increased disease severity by 30.45 units in CALS score (*p* < 0.001) compared to GG carriers. This result is in accordance with the results of a previous study by Gianni et al. (22). However, this is an unexpected finding. The *KLKB1*-rs3733402 variant locates in Apple domain 2 of the heavy chain where prekallikrein (PK) binds to high-molecular-weight kininogen (HMWK). The resulting reduced formation of the PK-HMWK complex interferes with optimal PK activation and reduces bradykinin formation and plasma kallikrein protection from inhibition by C1-INH.

Heterozygosity for *CPN1*-rs61751507 (c.533G>A, p.Gly178Asp) in carriers of missense *SERPING1* variants were independently associated with a 98% decrease in the probability of LTP (*p* = 0.017). In the past, *CPN1*-rs61751507 has been once associated with HAE when found in compound heterozygosity with a rare frameshift mutation in *CPN1*-exon 1 (17, 18). The effect of this variant observed in our study might be related to the substitution of the Gly^178^ residue of CPN the significance of which is underlined by the fact that it has been conserved in diverse species and is also conserved among most members of the human carboxypeptidase family.

Apart from our above findings that were confirmatory of previously described correlations between functional variants and parameters of the C1-INH-HAE severity, a series of novel correlations were uncovered in this study. The variants *F13B*-rs6003, *PLAU*-rs2227564, and *SERPINA1*-rs28929474 were found significantly correlated with the severity of C1-INH-HAE, independently of the *SERPING1* mutational status. Precisely, heterozygosity (GA) for *F13B*-rs6003 (c.344G>A, p.Arg115His) was correlated with decreased disease severity by 11.84 (*p* = 0.024) units of the CALS score. *F13B*-rs6003 has been considered benign concerning the FXIII subunit B deficiency. However, this variant has been characterized as a risk factor for venous thrombosis attributed to the substitution of Arg^115^ that prevents the dissociation between the A and B subunits of FXIII after activation by thrombin (14).

Carriage of T allele in homozygosity (TT) for *PLAU*-rs2227564 (c.422T>C, p.Leu141Pro) was found correlated with decreased disease severity by 13.67 units (*p* = 0.004) of the CALS score. Urokinase, encoded by *PLAU*, is an activator of plasminogen, which plays a significant role in the activation of the kinin-kallikrein system and the generation of bradykinin. The amino acid change p.Leu141Pro is located within the kringle domain of urokinase at the junction between two β-pleated sheets. The presence of the T allele does not appear to affect the activity of urokinase, but the zymogen containing Pro^141^ binds fibrin aggregates less efficiently than the one containing Leu^141^, suggesting a possibility of altered extracellular urokinase localization or stability (28).

The presence of the A allele of *SERPINA1*-rs28929474 (c.1096G>A, p.Glu366Lys) was correlated with increased disease severity by 80.16 units (*p* = 0.003) of CALS score. *SERPINA1*-rs28929474, commonly known as the Z allele of a_1_-antitrypsin (A1AT), is five times less effective than the normal M allele as an inhibitor of neutrophil elastase. It forms polymers in the lung that can be chemoattractants for neutrophils, thereby increasing inflammation (31–33), while it alters the global structural dynamics of A1AT (34). When found in a homozygous state, the Z allele is responsible for 95% of all clinical cases of A1AT deficiency, and in compound heterozygosity with the S allele, it is associated with 20–50% risk for emphysema (35, 36).

In addition to the above correlations detected between functional variants and disease severity, independently of *SERPING1* variation, further correlations were uncovered only in carriers of missense *SERPING1* variants. Heterozygosity (AT) for *SERPINA1*-rs17580 (c.863A>T, p.Glu288Val) was correlated with an 8-year earlier age at disease onset (*p* < 0.001) compared to AA carriers. *SERPINA1*-rs17580, commonly known as the S allele of A1AT, causes reduced cellular secretion of A1AT because the newly synthesized protein is degraded intracellularly before secretion (37). The S allele is not disease causing. Even homozygous carriers do not present the common expressions of A1AT deficiency. However, it represents a risk factor when in compound heterozygosity with the Z allele. Such compound heterozygotes are relatively frequent due to the high frequency of this allele. However, this compound heterozygosity was not detected among our patients.

Another functional variant related to disease severity in *SERPING1* missense carriers is *SERPINE1*-rs6092 (c.43G>A, p.Ala15Thr). Homozygous carriers (AA) of *SERPINE1*-rs6092 displayed a significantly higher mean age at disease onset by 8.4 years (*p* = 0.009). The variant has been previously characterized as likely benign for plasminogen activator inhibitor-1 (PAI-1) deficiency, but functional studies in a heterozygous patient showed activity at about 70%. Zhang et al. (44) suggested that the change from a hydrophobic non-polar amino acid (Ala) to a hydrophilic polar amino acid (Thr) in the hydrophobic core region (h-region) of the signal peptide of the protein may disturb its function.

Moreover, in the same group of C1-INH-HAE patients, heterozygous carriers (GA) of the gain-of-function mutation *F2*-rs1799963 (c^*^97G>A) had a significant decrease in the disease severity by 25.97 units in CALS score (*p* = 0.017) compared to GG carriers. According to Gehring et al. (15), this variant probably affects the generation of prothrombin. Finally, the *KLK1*-rs5515 (c.230G>A, p.Arg77His) variant was correlated with both the mean age at disease onset and the disease severity. Heterozygous carriers of this variant were presented with an 8.95-year later age at disease onset (*p* = 0.05) and with decreased disease severity by 16.79 units of the CALS score (*p* = 0.029). This variant had been characterized as a loss-of-function polymorphism resulting in reduced kallikrein activity. Slim et al. (20) detected 50–60% lower urinary kallikrein activity to carriers, while in studies concerning branchial artery function (21), which exhibited arterial dysfunction. Interestingly, this is an inverse correlation to what has been detected for the *KLKB1*-rs3733402 variant, as it is described above.

## Conclusion

Our study provides clear evidence that variants other than the *SERPING1* causal variants act as independent modifiers of C1-INH-HAE severity and could serve as possible prognostic biomarkers. The next step is the validation of detected correlations in a large cohort of patients. Such a study would best be performed by a global consortium of angioedema centers such as the ACARE network (56) to allow for the inclusion of a diverse and sizable population of clinically well-characterized patients. Enrolling large cohorts of patients could also allow examining the different effects possibly exerted by variants of modifier genes on carriers of different kinds of causal *SERPING1* mutations (nonsense, frameshift, large defects, etc.) with different impacts on C1-INH production, structure, and function. Furthermore, functional studies on the effect of these variants could shed light on missing parts of the pathogenesis of the disease. Finally, our results indicate that functional variants of genes involved in pathways other than contact activation system/kallikrein kinin system but recently recognized as participating in C1-INH-HAE pathogenesis (e.g., endothelial cells) should be investigated.

## Data Availability Statement

The data have been submitted to European Variation Archive (EVA) EMBL-EBI (https://www.ebi.ac.uk). The accessions associated with submission are: Project: PRJEB51008, Analyses: ERZ5253849.

## Ethics Statement

The studies involving human participants were reviewed and approved by the local institutional review boards, and written informed consent was obtained from each individual or an accompanying relative. Written informed consent to participate in this study was provided by the participants' legal guardian/next of kin.

## Author Contributions

FP performed genetic analyses, analyzed the results, and wrote the manuscript. GL performed genetic analyses and critically read the manuscript. MK analyzed the results. DC designed the study and collected clinical data. GP, FP, MMag, AV, MS, and KO collected clinical data. MZ, AS, MMau, MS, and HF critically read and commented on the manuscript. AG designed and supervised the study, critically read and revised the manuscript. All authors approved the submitted version of the article.

## Funding

This work was partly funded by the Hellenic Society of Angioedema. The funder had no role in the study design, data collection and analysis, decision to publish, or preparation of the manuscript.

## Conflict of Interest

The authors declare that the research was conducted in the absence of any commercial or financial relationships that could be construed as a potential conflict of interest.

## Publisher's Note

All claims expressed in this article are solely those of the authors and do not necessarily represent those of their affiliated organizations, or those of the publisher, the editors and the reviewers. Any product that may be evaluated in this article, or claim that may be made by its manufacturer, is not guaranteed or endorsed by the publisher.
