# Supplementary material for: Searching for Genetic Biomarkers for Hereditary Angioedema Due to C1-Inhibitor Deficiency (C1-INH-HAE)
Source: Front Allergy. 2022 Jul 7;3:868185. doi: 10.3389/falgy.2022.868185 (PMC9300820; doi:10.3389/falgy.2022.868185)
Supplement: Supplementary file 1 [file Data_Sheet_1.pdf]

Supplementary Table 1. Allele frequency of the 18 selected common SNPs in our cohort, compared to GMAF and EMAF.

SNP	Gene	Reference Allele	Homozygote Patients, %	Heterozygote Patients, %	Wild type Patients, %	Allele Frequency	GMAF	EMAF
rs1801020	***F12***	T	153/ 65.67%	73/ 31.33%	7/ 3.00%	81.33%	65.20%	75.66%
rs5985	***F13A1***	G	12/ 5.15%	77/ 33.05%	144/ 61.80%	21.67%	20.46%	25.33%
rs6003	***F13B***	G	173/ 74.25%	56/ 24.03%	4/ 1.72%	86.27%	88.22%	90.60%
rs1799963	***F2***	G	0/ 0.00%	12/ 5.15%	221/ 98.85%	2.58%	0.84%	1.25%
rs61751507	***CPN1***	G	0/ 0.00%	27/ 11.59%	206/ 88.41%	5.79%	4.23%	4.92%
rs669	***A2M***	A	27/ 11.59%	98/ 42.06%	108/ 46.35%	32.62%	30.98%	34.14%
rs5515	***KLK1***	G	0/ 0.00%	8/ 3.43%	225/ 96.57%	1.72%	3.12%	3.70%
rs3733402	***KLKB1***	G	71/ 30.47%	117/50.21%	45/ 19.31%	55.58%	54.02%	50.59%
rs72550870	***MASP2***	A	0/ 0.00%	26/ 11.16%	207/ 88.84%	5.58%	2.14%	3.23%
rs56378716	***MPO***	T	0/ 0.00%	4/ 1.72%	229/ 98.28%	0.86%	1.02%	1.35%
rs2227564	***PLAU***	T	138/ 59.23%	78/ 33.48%	17/ 7.30%	75.97%	74.83%	76.39%
rs17580	***SERPINA1***	A	0/ 0.00%	9/ 3.86%	224/ 96.14%	1.93%	2.33%	3.65%
rs28929474	***SERPINA1***	G	0/ 0.00%	3/ 1.29%	230/ 98.71%	0.64%	1.11%	1.84%
rs121912714	***SERPINA1***	A	0/ 0.00%	7/ 3.00%	226/ 97.00%	1.50%	0.05%	0.05%
rs6092	***SERPINE1***	G	1/ 0.43%	51/ 21.89%	181/ 77.68%	11.37%	9.52%	11.31%
rs5743708	***TLR2***	G	0/ 0.00%	12/ 5.15%	221/ 94.85%	2.58%	1.74%	2.81%
rs4926	***SERPING1***	G	13/ 5.58%	78/ 33.48%	142/ 60.94%	22.32%	22.20%	27.17%
rs28362944	***SERPING1***	T	2/ 0.86%	39/ 16.74%	192/ 82.40%	9.23%	2.91%	4.67%

Supplementary Table 2. Allele frequency of the 18 selected SNPs in C1-INH-HAE patients according to their nationality, compared to GMAF and EMAF.

SNP	Gene	Allele Frequency						GMAF	EMAF
		ALL (n=233)	BUL (n=19)	GR (n=31)	GER (n=23)	HUN (n=113)	POL (n=47)		
rs1801020	***F12***	81.33%	81.58%	87.10%	80.43%	79.65%	81.91%	65.20%	75.66%
rs5985	***F13A1***	21.67%	26.32%	14.52%	26.09%	21.68%	22.34%	20.46%	25.33%
rs6003	***F13B***	86.27%	84.21%	88.71%	89.13%	87.61%	80.85%	88.22%	90.60%
rs1799963	***F2***	2.58%	2.63%	4.84%	0.00%	3.54%	0.00%	0.84%	1.25%
rs61751507	***CPN1***	5.79%	2.63%	8.06%	2.17%	6.19%	6.38%	4.23%	4.92%
rs669	***A2M***	32.62%	39.47%	16.13%	32.61%	32.30%	41.49%	30.98%	34.14%
rs5515	***KLK1***	1.72%	5.26%	4.84%	2.17%	0.00%	2.13%	3.12%	3.70%
rs3733402	***KLKB1***	55.58%	47.37%	43.55%	47.83%	57.96%	64.89%	54.02%	50.59%
rs72550870	***MASP2***	5.58%	2.63%	4.84%	4.35%	6.19%	6.38%	2.14%	3.23%
rs56378716	***MPO***	0.86%	2.63%	1.61%	0.00%	0.88%	0.00%	1.02%	1.35%
rs2227564	***PLAU***	75.97%	78.95%	91.94%	78.26%	70.80%	75.53%	74.83%	76.39%
rs17580	***SERPINA1***	1.93%	0.00%	0.00%	0.00%	3.54%	1.06%	2.33%	3.65%
rs28929474	***SERPINA1***	0.64%	0.00%	0.00%	2.17%	0.00%	2.13%	1.11%	1.84%
rs121912714	***SERPINA1***	1.50%	2.63%	0.00%	0.00%	2.65%	0.00%	0.05%	0.05%
rs6092	***SERPINE1***	11.37%	13.16%	6.45%	8.70%	12.83%	11.70%	9.52%	11.31%
rs5743708	***TLR2***	2.58%	0.00%	0.00%	2.17%	3.54%	3.19%	1.74%	2.81%
rs4926	***SERPING1***	22.32%	21.05%	19.35%	23.91%	19.91%	29.79%	22.20%	27.17%
rs28362944	***SERPING1***	9.23%	5.26%	4.84%	2.17%	13.72%	6.38%	2.91%	4.67%
